# A first experience of transduction for differentiated HepaRG cells using lentiviral technology

**DOI:** 10.1038/s41598-019-49402-8

**Published:** 2019-09-09

**Authors:** Adeline Pivert, Caroline Lefeuvre, Cong-Tri Tran, Claude Baillou, David Durantel, Hélène Le Guillou-Guillemette, François M. Lemoine, Françoise Lunel-Fabiani, Alexandra Ducancelle

**Affiliations:** 1 Laboratory of Virology, University Hospital & LUNAM University and HIFIH laboratory, UPRES EA 3859, SFR 4208 Angers, France; 2 Sorbonne Université, Inserm, CNRS, Centre d’Immunologie et Maladies Infectieuses (CIMI-Paris), Paris, France; 3 Cancer Research Center of Lyon (CRCL), INSERM, U1052, CNRS, University of Lyon, UMR_5286, LabEx DEVweCAN, Lyon, France

**Keywords:** Microbiology techniques, Hepatitis B

## Abstract

Currently, there is a lack of systems for studying the role of hepatitis B viral proteins, such as HBeAg and HBcAg, on liver injury. It is necessary to develop an original tool in order to clarify the role of these viral proteins in hepatic stellate cell activation, and to understand the molecular mechanisms of liver injury. HepaRG are the most reliable hepatocyte-like cells for studying liver functions or disorders. In this paper, we demonstrate that the transduction of differentiated HepaRG (dHepaRG) cells can be performed successfully using lentiviral particles. The production of a functional Green Fluorescent Protein (GFP) assessed by Fluorescence Activated Cell Sorting and fluorescence microscopy is up to 16% of GFP positive cells using a multiplicity of infection (MOI) of 2.4. We demonstrate that this technology can allow the stable expression of GFP during the long lifecycle of the cell (up to four weeks after the cell’s passage). With this innovative tool, we aim to express viral proteins such as HBeAg or HBcAg in dHepaRG cells. The preliminary results of this work shows that HBeAg can be efficiently produced in dHepaRG cells and that increased MOI allows a better production of this protein. Our future objective will be to study the role of HBc and HBe proteins on the induction of hepatic fibrosis.

## Introduction

Liver fibrosis is a common complication of chronic hepatitis B. The pathogenesis of liver fibrosis involves significant accumulation of fibrillar collagens and other extracellular matrix proteins. Several cell types are implicated in the pathogenesis of liver fibrosis but hepatic stellate cell (HSC) activation is substantial in liver fibrosis^1^. The role of HBV, and/or its specific viral proteins in HSC activation and liver fibrosis, remains to be clarified. Recent data support the hypothesis that the HBx protein is closely related to the development of hepatocellular carcinoma (HCC)^2^, as well as its implications for HSC activation *in vitro*^3^. However, the fibrotic role of HBe and HBc proteins in liver fibrosis is not well described. In fact, the experimental models used to investigate the interaction of HBV proteins with HSC, and the functions of viral proteins have significant limitations. Primary human hepatocytes (PHH) lose liver functions such as drug metabolism or polarity during culture. The method of preparation is time consuming, and human genetic diversity involves heterogeneous material for quality and limited availability. Although many different liver-derived cell lines exist, HepaRG is an efficient *in vitro* model used for multiple applications: the study of absorption, distribution, metabolism and excretion, toxicity testing applications (hepatotoxicity, virology, etc.), BioArtificial Livers and transgenic liver humanized mice. Progenitor HepaRG cells (pHepaRG) can be differentiated in hepatocyte-like cells (named differentiated HepaRG or dHepaRG) after exposure to DMSO and hydrocortisone, two well-known differentiation inducers^4^. dHepaRG cells, showing a typical aspect of hepatocytes clustered in small colonies, offer similar molecular characteristics to PHH, including morphology, nuclear receptors, expression of key metabolic enzymes, and drug transporters^4,5^.

Lentiviral technology is an efficient method for mediating gene transfer to a large number of cell types, using vectors usually derived from the human immunodeficiency virus (HIV) genome. Integration of the transgene into the host genome provides a prolonged and stable expression of the vector-containing protein. The lentiviral expression system is able to overcome plasmid transfection experimental requirements, such as the cell cycle stage (quiescent or dividing cells) or the transfection permissiveness of each cell type. Furthermore, there is no cytopathogenic effect with this technology^6,7^.

Many authors have described the lentiviral transduction of pHepaRG cells to obtain a stable expression of cellular genes in hepatocyte. After the transduction of pHepaRG, cell differentiation with DMSO, another treatment was performed in order to overexpress a receptor, or to silence a mitochondrial component^8–10^. Another recent study used lentiviral technology to transduce pHepaRG and to express a Farnesoid receptor enabling study of the impact of this protein expression on the infection, replication and persistence of HBV^11^. Moreover, Gripon *et al*. demonstrated that differentiation state of HepaRG had an impact on the liver-specific mRNAs expression, such as the mRNA for albumin. Low or undetectable amounts of mRNAs were observed in proliferating HepaRG cells, whereas at confluence for one month, albumin and aldolase B mRNAs became highly expressed^4^.

In this context, we aimed to transduce dHepaRG in order to produce HBc and HBe proteins in cells which express specific hepatic functions such as normal human hepatocytes. To our knowledge, there are no published data concerning HBV protein production using dHepaRG transduction using lentiviral particles.

As the transduction of dHepaRG cells with lentiviral particles is not yet well described in the literature, this paper reports on the transduction of dHepaRG cells using lentiviral particles to produce proteins. First, the Green Fluorescent Protein (GFP) was used as protein control to valid the production of lentivirus particles and the transduction of dHepaRG. Secondly, the HBeAg was produced using the transduction of dHepaRG by lentivirus particles containing HBV precore/core gene to optimize the dHepaRG transduction efficiency. Our future objective will be to study the role of HBc and HBe proteins on the induction of hepatic fibrosis with the development of a cellular co-culture model using dHepaRG and hepatic stellate cells.

## Results

### Lentiviral particle titration

For lentiviral particles containing GFP, three assays of lentiviral production were performed and p24 Ag was measured after concentration by centrifugation. The mean concentration of p24 in viral supernatants was 5.83 ± 3.65 × 10^5^ pg/mL (range 3.24 × 10^5^ to 1 × 10^6^ pg/mL). For lentiviral particles containing precore/core gene, three assays of lentiviral production were performed and p24 Ag was measured after concentration by centrifugation. The mean concentration of p24 in viral supernatants was 4.47 ± 3.87 × 10^5^ pg/mL (range 1.2 × 10^5^ to 9.9 × 10^5^ pg/mL).

**Figure 1:**
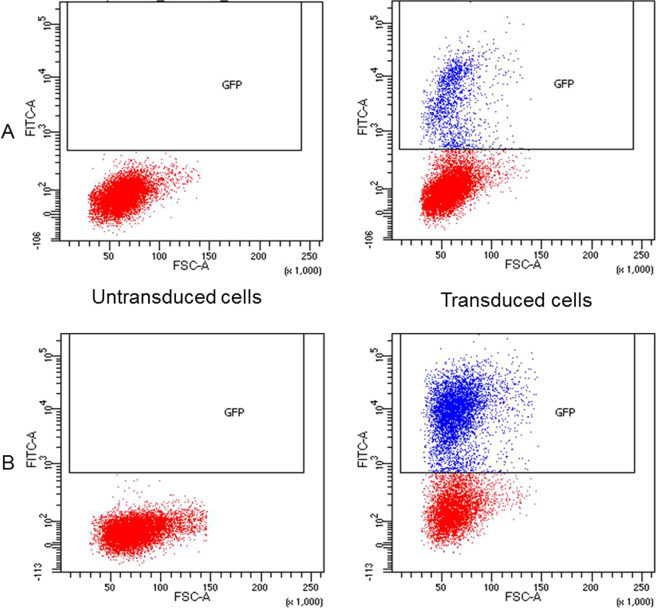
Quantification of GFP expression by flow cytometry in HEK 293T cells following lentiviral transduction in order to establish the functional titer of lentiviral particles. The horizontal axis represents the forward scatter size (FSC-A) where an increased signal may indicate an increase in cell size or budding. The vertical axis indicates the GFP fluorescence intensity (FITC-A). Blue dots represent GFP-positive cells. Red dots represent GFP-negative cells. The threshold for GFP positivity was determined according to negative control (non-transduced cells). The concentrated viral supernatant titer was calculated using various supernatant dilutions, an average GFP-positive cell count of 15.8%, corresponding to a 10^−1^ dilution of the viral supernatant (**A**). The percentage of GFP-positive cells using 10 μL of concentrated viral supernatant is 52.8% (**B**). Four assays were performed with the 10^−1^ dilution of the viral supernatant: the mean titer was 1.7 × 10^7^ ± 1.05 × 10^7^ TU/mL.

For lentiviral particles containing GFP, functional titration was carried out using FACS (Fluorescence Activated Cell Sorting). Twenty-four hours after seeding, approximately 2 × 10^5^ target cells were counted. The threshold of fluorescence for FACS results was determined according to the negative control represented by non-transduced cells. The cells expressing GFP showed significant fluorescence of FITC. The mean GFP titer following lentiviral transduction was 1.7 × 10^7^ TU/mL (Transducing Units, TU) (n = 4). The concentrated viral supernatant titer was calculated with an average GFP-positive cell count of 15.8%, corresponding to a 10^−1^ dilution of the viral supernatant (Fig. 1A). Using a concentrated viral supernatant, 52.8% of the cells were GFP-positive (Fig. 1B).

**Figure 2:**
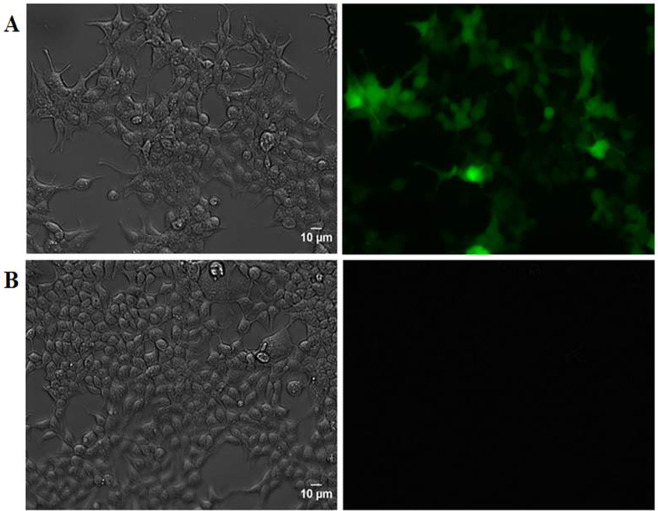
Phase contrast and fluorescence microscopy of 293T cells after transduction with lentiviral particles containing the GFP gene. Cells were transduced with 10 μL of concentrated viral supernatant and emitted a fluorescent signal (**A**); Non-transduced cells were used as negative control (**B**). The scale bar represents 10 μm (**A,B**).

Moreover, expression of GFP observed in fluorescence microscopy confirmed that our lentiviral transduction was effective (Fig. 2).

Using the qPCR, the quantitation of cells was 49,000 for the well containing 1 µL of the supernatant of lentiviral particle containing GFP, according to the GAPDH detection compared to the standard curve. 219,200 copies of lentiviral genome were quantified for the same well. For lentiviral particles containing GFP, the proviral DNA titer calculated with qPCR was 1.3 × 10^9^ TU/mL (*n* = *1*). For lentiviral particles containing precore/core gene, the proviral DNA titer calculated with qPCR was 1.3 × 10^9^ TU/mL (*n* = *2*) (Table 1).

**Table 1:** Determination of lentiviral titers by qPCR.

	LVP GFP		LVP HBe
Volume of supernatant	10 µL	1 µL	10 µL
GAPDH average (copies/extract)	6.76 × 10^4^	4.9 × 10^4^	9.78 × 10^5^
LV2 average (copies/extract)	1.18 × 10^6^	2.19 × 10^5^	4.2 × 10^7^
Lentiviral copy/cell	48.3	6.5	42.9
Titer (TU/mL)	9.7 × 10^8^	1.3 × 10^9^	1.3 × 10^9^

TU = Titer Units, LVP = lentiviral particule.

### Transduction of dHepaRG cells to produce GFP protein

**Figure 3:**
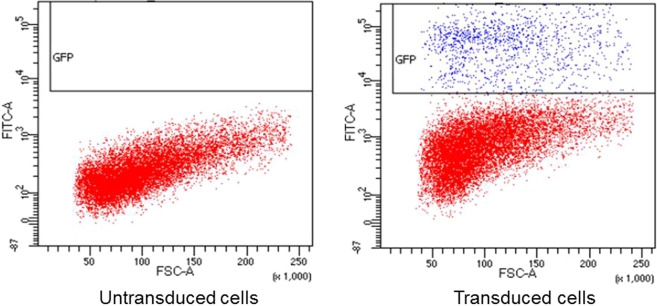
Quantification of GFP expression by flow cytometry in dHepaRG cells 10 days after lentiviral transduction. The horizontal axis represents the forward scatter size, (FSC-A) where an increased signal may indicate an increase in cell size or budding. The vertical axis indicates the intensity of GFP fluorescence (FITC-A). Blue dots represent the cells expressing GFP. Red dots represent the cells that do not express GFP. The GFP positivity threshold of the cells was determined according to negative control (non-transduced cells). The percentage of GFP-positive cells at an MOI of 2.4 is 10%.

**Figure 4:**
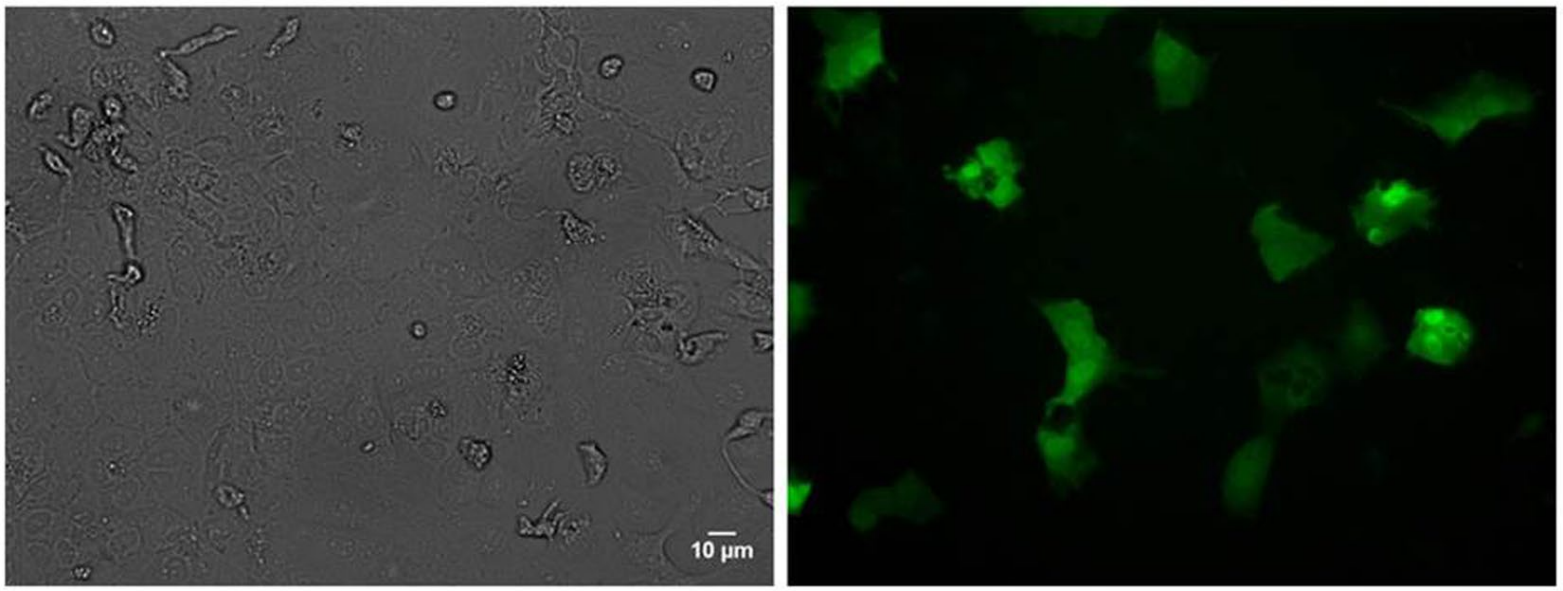
Phase contrast and fluorescence microscopy of dHepaRG cells expressing GFP 10 days after lentiviral transduction. Cells were transduced with viral supernatant at a MOI of 2.4. The scale bar represents 10 μm.

Ten days after the lentiviral transduction of dHepaRG cells, the percentage of GFP-positive cells was 10% (Multiplicity of Infection, MOI 2.4) when observed using FACS (Fig. 3). A second assay was conducted with 16% of GFP positive cells. The threshold of fluorescence for FACS results was determined according to the values observed with negative control that corresponded to non-transduced cells. Expression of GFP was also confirmed by microscopy (Fig. 4).

**Figure 5:**
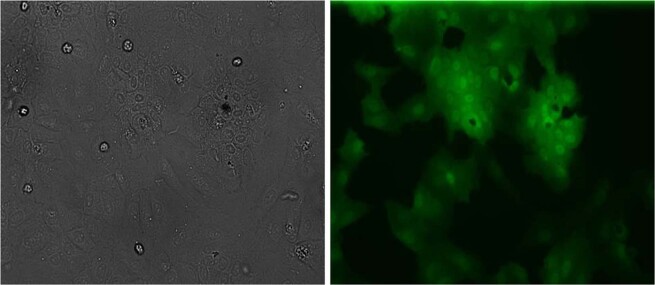
Phase contrast and fluorescence microscopy of dHepaRG cells expressing GFP 4 weeks after lentiviral transduction. Cells were transduced with viral supernatant at a MOI of 2.4. The scale bar represents 10 μm.

Four weeks after the lentiviral transduction of dHepaRG cells and cell passage, numerous GFP-positive cells were observed by microscopy. We observed that fluorescence was localized in the hepatocyte-like cells (Fig. 5).

### Transduction of dHepaRG to produce HBe protein

**Figure 6:**
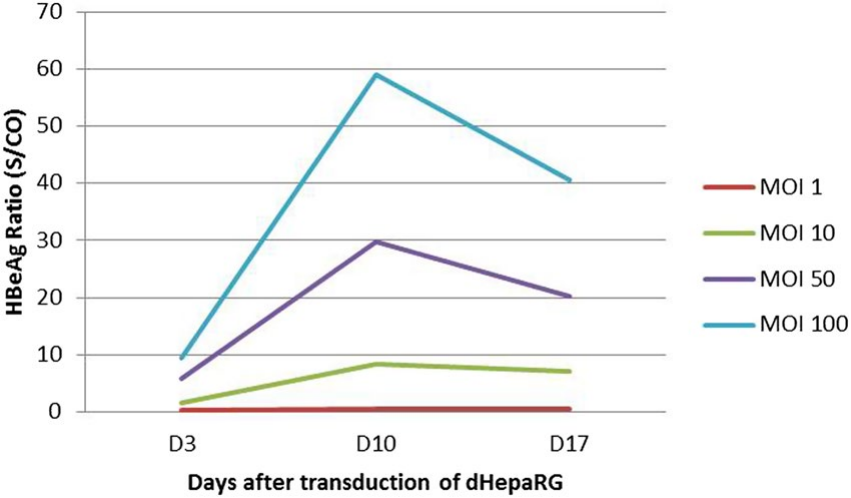
HBeAg ratio measured by CMIA for the different MOI tested at days 3, 10 and 17 after the transduction of dHepaRG (n = 1).

For MOI 1, no production of HBeAg was detected at days 3, 10 or 17 after dHepaRG transduction. For the MOI 10, 50 and 100 HBeAg were detected at day 3 and the ratio increased until day 10. At day 17, a decrease in the HBeAg ratios was observed for MOI 100 and 50 after the addition of DMSO to the culture medium (Fig. 6).

The values of HBeAg ratio were lesser than expected but MOI 100 allowed a better production of HBeAg than the others. When we analyzed the HBeAg ratio according to the days after dHepaRG transduction, the MOI 100 at day 10 appeared to have the higher HBeAg ratio.

## Discussion

The main difficulties in producing proteins using gene transfer technology are the expression rates and long-term stability. Most gene transfer methods that relate to HepaRG have used synthetic vectors such as lipofection and electroporation techniques. In fact, Laurent *et al*. explained that plasmid DNA and formulations with neutral co-lipids, the lipophosphoramidate compounds KLN47 and BSV10, allowed the expression of the GFP in ~50% of adherent progenitor HepaRG cells, while electroporation targeted GFP expression in ~85% of both progenitor and differentiated cells in suspension^12^. With regard to the lipofection method, the reproducibility and low yield of intracellular penetration are unsatisfactory. Moreover, Demazeau *et al*. reported that egg phosphatidylcholine/Diether-NH2-based liposomes enabled an efficient transfection in both HEK 293T and proliferating pHepaRG cells, with similar efficacies with our technology: 25–90% and 16% respectively^13^. However, a high cell death rate and laborious development was reported for the electroporation method. The effective transduction of proliferating and differentiated HepaRG cells is also possible with recombinant baculoviruses carrying the genome of interest^7^. The authors do not described the efficiency of transduction. Furthermore, Zhao *et al*. demonstrated that the adenoviral vector-mediated transduction yields viral titers 10 times higher than the transient transfection approach^14^. For recombinant baculoviruses and adeno-associated vectors, protein expression over time is limited because the viral genome is not integrated into the cellular genome, therefore giving rise to transient expression. None of these technologies allow a stable expression of the transgene in dividing cells. During cells division, the transgene can’t be maintained without a pressure selection, because it is not integrated into the cell genome. So, according to the published data, lentiviral technology has been chosen in our project to get a stable expression of interest protein, even if dividing cells, and with low cell death rate.

The highlight of this work is the validation of dhepaRG transduction to produce interest proteins. Firstly, before conducting the dHepaRG transduction, an evaluation of the lentiviral titer was required in order to determine whether the lentiviral particles produced were effective. Various methods of functional and non-functional lentiviral titration have been described in the literature, including p24 antigen ELISA, RNA titers, FACS and quantification of proviral DNA by qPCR. In our report, lentiviral particle production was reproducible according to the weak standard deviation observed between our assays. The calculated RNA (data not shown) and p24 titers as the quantification of the proviral DNA were consistent with those described in the literature, confirming the acquisition of the lentiviral technology at our laboratory^15^.

Secondly, dHepaRG cell transduction using recombinant defective lentiviral particles has been performed in order to express GFP or viral proteins. According to our results obtained by fluorescence microscopy and FACS, the lentiviral technology seems to be efficient in dHepaRG cells: GFP expression is stable and prolonged. Indeed, expression of the GFP was observed in up to 16% of dHepaRG cells (MOI 2.4) 10 days after infection and remained stable for at least four weeks after a passage of dHepaRG cells. However, the expression of GFP appears over 10 days after transduction, as we can see many fluorescent islet cells by microscopy. Lentiviral vectors are efficient vehicles for stable gene transfer into hepatocytes, and their capacity to transduce a large scale of cells is a great advantage. In our experience, dHepaRG cells appear to be easy to transduce with lentiviral particles, according to the classical constraints of cellular culture despite a poor effectiveness rate (up to 16%, MOI 2.4). As previously described, the use of a higher MOI may be improving the efficiency of transduction. The authors show that MOI 100 for baculovirus and 200 to 1000 for adenovirus allow a higher efficiency of transduction^7,14^. In order to verify this hypothesis, dHepaRG transduction with lentiviral particles containing precore/core gene of HBV was performed with several MOI from 1 to 100. Our results confirmed that the relative quantity of HBeAg increased when the MOI rose too. We also demonstrated that DMSO had an adverse effect on the production of HBeAg, probably inducing cell mortality. These experiments on viral protein allowed us to conclude on three hypotheses: (i) we were able to transduce dHepaRG with lentiviral particles which contained viral transgene, (ii) the interest protein was efficiently produced and can be easily detected and (iii) a MOI of 100 allowed better production of HBeAg. Concerning the adverse effects of DMSO, further experiments are in progress at our laboratory for studying of the impact of varying percentages of DMSO in the culture medium at day 4 after transduction.

However, several limitations can be identified in our work. First, the low efficiency of transduction of dHepaRG with the GFP gene can be explained by the weak MOI tested (2.4). The preliminary results were obtained with the transduction of GFP, in order to validate the capacities of lentiviral particles to transduce dHepaRG. The transduction efficiency with a large range of MOI has been assessed with HBeAg in order to optimize the lentiviral transduction of dHepaRG for HBeAg production and not GFP. Second, we recognize that more experiments are request to perform relevant statistics which are missing in the present work. Third, this tool can be improved if the sequence of GFP or another reporter gene was integrated in the vector plasmid used for the lentiviral particles construction. This characteristic makes it easier to determine the proportion of transduced HepaRG cells, which was not possible with the transduction of lentiviral particles containing only the precore/core transgene.

Therefore, this paper is the first to conclude that dhepaRG transduction using lentiviral technology could be an innovative tool for producing HBeAg. The functions of HBe and HBc proteins in the occurrence of liver disease remain controversial. Zan *et al*. found that HBeAg induced the secretion of TGF-β in rat hepatic stellate cells^16^. Some authors have demonstrated that HBc protein promotes HSC proliferation and increases the mRNA levels of profibrotic genes^17^, whereas others demonstrated the expression of HBc sensitized hepatocytes to the TNF factor inducing apoptosis and liver injury during hepatitis B virus infections^18^. A recent study reveals a novel mechanism involving HBc protein in HBV-related hepatocarcinogenesis, indicating that the HBc protein may promote HCC^19^. Conversely, other findings conclude that the precore protein has no significant fibrotic impact^20^.

Indeed, further studies are necessary to understand the role of HBe and HBc proteins in hepatic stellate cell activation and liver injury. Our team would like to develop molecular tools for studying the impact of HBc and HBe proteins on the induction of hepatic fibrosis. After dHepaRG transduction with the lentiviral particles containing the transgenes of interest to produce the HBc and HBe proteins, we should be able to analyze the behavior of the HSC by measuring the production of pro-fibrotic cytokines such as TGF-β, PDGF and α-SMA1 by qPCR in cell supernatants.

Therefore, we conclude that dHepaRG transduction using lentiviral technology is an innovative tool for the efficient production of proteins. Furthermore, dHepaRG transduction could be useful for many applications, such as producing other specific HBV proteins and studying hepatic fibrogenesis.

## Materials and Methods

### Cell lines

HEK 293T (ATCC® CRL-3216 ™) cells used for transfection and transduction, are human embryonic kidney cells transformed with the SV40 T-antigen. HEK 293T cells were maintained in Dulbecco’s Modified Eagle’s Medium (DMEM, Gibco®), supplemented with 10% fetal calf serum (Fetal Bovine Serum FBS, Gibco®), 2 mM L-glutamine (Gibco®) in a humidified incubator containing 5% CO_2_ in air at 37 °C.

The HepaRG cell line (UMR INSERM 1052 CNRS 5286-Cancer Research Center, Lyon) is maintained in William’s Medium E medium (Gibco®) supplemented with 10% FetalClone® II Serum (HyClone™), 2 mM GlutaMAX™ (Gibco®), 5 μg/mL human insulin (Sigma-Aldrich®), 50 μM hydrocortisone (SERB laboratory), 1.8% DMSO (Sigma-Aldrich®) and 50 U/mL penicillin/streptomycin in a humidified incubator containing 5% CO_2_ in air at 37 °C. A minimum of four weeks’ culture in this medium is required to obtain differentiated HepaRG cells.

### Lentiviral particle production and titration

Lentiviral particles containing the GFP reporter gene or the HBV precore/core gene were produced as previously described^21^. The three plasmids were co-transfected into HEK 293T cells with 12 µg of a mixture containing the plasmid vector pENG1 EF1α-GFP or precore/core (5.6 μg), the encapsidation plasmid pCMV-9 (4.4 μg) and the envelope plasmid VSV- G (2 μg). The FuGENE® 6 Transfection Reagent (Promega)/Opti-MEM™ (Gibco®) was used following a FuGENE/DNA ratio of 3:1. The culture supernatant was harvested, purified and concentrated by ultracentrifugation (2 hours; 50,000 g).

#### HIV-1 Gag (p24) measurement

The p24 concentration in viral supernatants was determined by the certified p24 immunoassay ELISA (VIDAS^®^ HIV P24 II, bioMérieux) according to the manufacturer’s instructions. Dilutions with 10^−3^ and 10^−4^ of the concentrated viral supernatants were tested. The dilutions were carried out in DMEM medium. The titer is expressed in pg p24/mL.

### Efficiency of infection with lentiviral particles containing the GFP gene

#### GFP titer: flow cytometry

HEK 293T cell transduction was performed with lentiviral particles containing the reporter gene GFP. One hundred thousand HEK 293T cells were seeded per well in a 12-well plate. Twenty-four hours later, the cells were counted and dilutions with 1, 10^−1^, 10^−2^, 10^−3^, 10^−4^ and 10^−5^ of pENG1 EF1α-GFP vector supernatants were prepared in DMEM medium supplemented with 10% FBS. Then, 10 µL of each dilution was added to 500 µL of DMEM medium supplemented with 10% FBS and 8 μg/mL Polybrene® (Sigma-Aldrich®) and incubated with cells.

Seventy-two hours after the addition of the viral supernatant, the expression of GFP was observed through an inverted fluorescence microscope (Leica DMI6000 B).

Then, cells were collected after adding 0.05% trypsin-EDTA, centrifuged for 5 minutes with 500 g at 4 °C and the cell pellet was suspended in 1% paraformaldehyde (Sigma-Aldrich®) in order to fix cells and inactivate viral particles. A further centrifugation with 500 g for 5 minutes was carried out and the cell pellet was suspended in 1 mL of cold Phosphate Buffered Saline 1X. A sample was analyzed for fluorescence with a FACSCanto™ BD flow cytometer (BD Biosciences). The titer was expressed in TU/mL and calculated using the following equation: (number of target cells 24 hrs after seeding) × (virus dilution factor) × (percentage of GFP-positive cells)/(volume of viral supernatant added in mL). GFP titers were calculated with an average of FACS values at vector dilutions (in duplicate) corresponding to 1–20% of GFP-positive cells in FACS analysis. Selecting values in this range decreases the risk of analyzing cells with multiple copies of the vector, which would underestimate the titer. Measurement of GFP in FACS after limiting dilution in the cell culture provided data of transduction efficiency and the remaining functional viral titer.

#### Proviral DNA titer

For determining DNA titers, genomic DNA from transduced HEK 293T cells was isolated four days after transduction using the NucleoSpin^®^ Tissue kit, (Macherey-Nagel) according to the manufacturer’s recommendations. Five µL of genomic DNA from non-transduced cells was used for negative controls. All reactions were carried out in duplicate. For real-time PCR, the aforementioned primers FPLV2 and RPLV2 and LV2 probe were used^14^. Five µL of DNA was mixed with 20 µL of a Master Mix containing 1X TaqMan™ Universal PCR Master Mix (ThermoFisher Scientific), 320 nM of forward (FPLV2) and reverse (RPLV2) primer, 200 nM of the lentiviral probe (LV2). Amplification was performed using one 95 °C cycle for 10 minutes and 30 cycles at 95 °C for 30 seconds and 60 °C for 2 minutes. Vector plasmid DNA amplification was performed using concentrations ranging from 1.23 × 10^7^ copies/µL to 1.23 × 10^3^ copies/µL to generate a standard curve. We used the GAPDH gene to normalize the qPCR results. The vector DNA titer was determined by considering the following parameters according to the protocol: number of cells plated and infected (number of cells counted in day 2), lentiviral copy number per cell, and volume of used lentivirus. The lentiviral copy number per cell was determined by the following equation: (copy number LV2)/(copy number GAPDH) × 2. The DNA titer (TU/mL) was calculated according to the following formula: (primary number of cells counted in day 2) × (lentiviral copy number per cell) × (dilution factor)/(volume of used lentivirus in mL).

### dHepaRG cells transduction to produce GFP

After four weeks of culture in the presence of DMSO and hydrocortisone, the differentiated HepaRG cells were transduced by lentiviral particles containing the GFP transgene to a MOI of 2.4. The MOI tested was deduced using the following equation: (viral titer in TU/mL) × (volume of viral supernatant added to cells in mL)/(number of seeded cells). At day 0 of transduction, the medium of approximately 2.10^5^ HepaRG cells was changed by a solution containing viral supernatant, 8 μg/mL of Polybrene® and the usual medium. After the addition of viral supernatant, cells were incubated at 37 °C with CO_2_. The kinetics of GFP expression by lentivirally infected cells was observed by fluorescence microscopy at different times: 48, 72, 96 hours and nine days after transduction. The test was carried out in duplicate. The percentage of GFP-positive transduced cells was determined by FACS analysis ten days after transduction.

### dHepaRG cells transduction to produce HBe protein

Lentiviral particles with HBeAg transgene were produced and tittered using the same procedure as GFP. dHepaRG cells were transduced at several MOI (1, 10, 50 and 100) in order to compare the quantity of HBeAg expressed. At day 0 of transduction, 1.2 × 10^6^ HepaRG cells were counted. A single test can be performed in account of the quantity of lentiviral particles produced. The kinetics of HBeAg expression by transduced cells was analyzed with chemiluminescence (CMIA, Abbott) at different times: days 3, 10 and 17 after transduction. At day 10 after transduction, DMSO was added to the culture medium in order to conserve the differentiated status of HepaRG.
