# Publisher Correction: A first experience of transduction for differentiated HepaRG cells using lentiviral technology

**DOI:** 10.1038/s41598-021-97243-1

**Published:** 2021-09-06

**Authors:** Adeline Pivert, Caroline Lefeuvre, Cong-Tri Tran, Claude Baillou, David Durantel, Hélène Le Guillou-Guillemette, François M. Lemoine, Françoise Lunel-Fabiani, Alexandra Ducancelle

**Affiliations:** 1 Laboratory of Virology, University Hospital & LUNAM University and HIFIH Laboratory, UPRES EA 3859, SFR 4208, Angers, France; 2 Sorbonne Université, Inserm, CNRS, Centre d’Immunologie et Maladies Infectieuses (CIMI-Paris), Paris, France; 3 Cancer Research Center of Lyon (CRCL), INSERM, U1052, CNRS, University of Lyon, UMR_5286, LabEx DEVweCAN, Lyon, France

Correction to: *Scientific Reports* 10.1038/s41598-019-49402-8, published online 09 September 2019

The original PDF version of this Article omitted Figure 6. This error has now been corrected in the PDF version of the Article; the HTML version was correct from the time of publication.

